# Exogenous melatonin on photosynthesis and physiological characteristics in maize under drought stress

**DOI:** 10.1186/s12870-026-08100-0

**Published:** 2026-01-28

**Authors:** Hafeez Noor, Kuang Sheng

**Affiliations:** 1 College of Plant Protection, Shanxi Agricultural University, Jinzhong, 030801 China; 2 Biotechnology and germplasm resource institute, Yunnan Academy of Agricultural Sciences, Kunming, 650000 China

**Keywords:** Melatonin, Maize, Drought stress, Photosynthetic capacity, Antioxidant system

## Abstract

**Background:**

Drought stress severely limits maize production in Northwest China. Although melatonin is known to enhance plant stress tolerance, its specific mechanisms in maize have not been fully elucidated. To address this, we investigated whether melatonin improves drought tolerance using two contrasting maize genotypes: drought–tolerant (SD–609) and drought–sensitive (SD–902). A experiment was conducted under drought stress with and without melatonin application, in which we have analyzed photosynthetic parameters, antioxidant enzyme activities, oxidative stress markers, key components of CO₂ assimilation, and dry matter accumulation.

**Results:**

Under drought stress, melatonin application significantly enhanced photosynthetic performance by improving dark reactions (upregulation of CO₂ assimilation enzymes and genes). It concurrently activated the antioxidant system (SOD, CAT, POD, and the AsA–GSH cycle), leading to a marked reduction in H₂O₂ and MDA levels. These synergistic improvements resulted in a significant increase in dry matter production. The drought–tolerant genotype (SD = 609) exhibited a more pronounced response to melatonin across these parameters than the sensitive genotype (SD = 902).

**Conclusions:**

This study demonstrated the co–activation of key photosynthetic and antioxidant pathways. The stronger effect in the drought–tolerant genotype suggests an interaction with inherent genetic capacity. These findings provide a crucial theoretical basis for the application of melatonin as a potential agrochemical to enhance crop resilience in water–limited environments.

**Supplementary Information:**

The online version contains supplementary material available at 10.1186/s12870-026-08100-0.

## Introduction

As a global source of food, feed, and industrial raw materials, maize (*Zea mays* L.) yield stability is critical for ensuring global food security [1]. Drought stress not only affects the geographical distribution of plants and limits crop yields but also poses a threat to global food security. Drought inflicts the greatest agricultural damage due to natural disasters, causing an estimated 50% yield loss, a threat magnified by the fact that 43% of global croplands are in arid and semi–arid regions [2]. Approximately 48% of China’s land area is located in arid and semi–arid regions, which makes crop production highly susceptible to drought stress [3]. At the cellular level, drought impairs plants by disrupting key metabolic pathways and inflicting structural damage, including to cell membranes and the fine architecture of organelles [4], inhibition of leaf growth, stem elongation, root cell proliferation, reduction in stomatal conductance, and significant decrease in photosynthetic rate [5]. Drought affects the growth and development of crops, reduces the accumulation of crop biomass, and thereby affects yield formation [6]. Stomatal limitation refers to a plant’s adaptive strategy under drought stress, whereby stomatal closure or reduced aperture minimizes water loss, though it also restricts CO₂ uptake [7]. Non–stomatal limitation describes the decline in photosynthetic capacity resulting from direct biochemical and structural damage under drought stress, including impaired thylakoid membrane integrity, disrupted photosystem function, inhibited electron transport, and decreased activity of Calvin cycle enzymes [8]. Under different varieties, different degrees, and different types of drought stress, the degree to which drought stress inhibits photosynthesis in plants varies [9]. Drought stress in maize impairs the photochemical efficiency of PSII, leading to a decrease in photochemical quenching (qP) and a corresponding increase in non–photochemical quenching (NPQ) [10]. Photosynthetic system due to oxidative stress and a decrease in activity, and maize dissipates excess light energy through a heat dissipation mechanism to alleviate oxidative damage to the photosynthetic system [11]. An increase in drought stress inhibits photosynthetic electron transfer activity and light quantum utilization efficiency of maize leaves [12]. Beyond its effects on photochemical efficiency, drought stress has been shown to inhibit electron transport along the photosynthetic electron transfer chain, affecting both PSII and PSI donor–side activity [13, 14]. The process of regenerating PEP from pyruvic acid is catalyzed by PPDK located in the chloroplasts of mesophyll cells. Key to stress tolerance, enzymes like Carbonic Anhydrase (CA) support critical functions including ion and pH homeostasis, carboxylation/decarboxylation catalysis, and chloroplastic CO₂ supply [15], which is conducive to maintaining the stability of photosynthetic carbon metabolism under stress conditions. Under salt, high temperature, and drought stress conditions, the activity of cotton CA changes according to changes in photosynthetic rate [16]. Phosphoenolpyruvate carboxylase (PEPC) is a key enzyme involved in CO_2_ fixation in maize. Adverse conditions, such as drought stress, cold damage, and salt stress can induce an increase in the expression levels of the PEPC gene [17]. Under drought stress conditions, overexpression of the maize PEPC gene in wheat increases the expression of photosynthetic enzyme genes PEPC, NADP–ME, PPDK, and the large subunits rbcl and rbcs of RuBPc; increases the activity of photosynthetic enzymes PEPC, NADP–ME, PPDK, and RuBP carboxylase in wheat; and enhances the photosynthetic capacity of wheat under drought stress [18]. PPDK is a rate–limiting enzyme in the C4 plant photosynthetic pathway. Transferring the high–efficiency genes ZmPPDK and ZmPEPC in maize significantly enhances the drought resistance of Arabidopsis [19]. Under drought stress, the photosynthetic electron transport chain is disrupted, resulting in the inability of plants to dissipate excess energy. This leads to overproduction of reactive oxygen species (ROS) such as singlet oxygen, hydroxyl radicals, superoxide anion radicals, and hydrogen peroxide. Chloroplasts are the primary sites of ROS generation, where superoxide and hydrogen peroxide are produced via the Mehler reaction, and singlet oxygen is formed through photoprotective processes. Consequently, the photosynthetic apparatus is a key target for ROS–induced oxidative damage [20]. The primary and secondary quinone receptors of PSII, plastocyanin (PQ), the cytochrome complex, plastocyanin (PC), and the PSII reaction center [21]. Excessive ROS can inactivate biomolecules and cause oxidative damage to membranes and other macromolecules, such as photosynthetic pigments, proteins, lipids, and nucleic acids, thereby leading to photooxidation and photoinhibition [20, 22]. Research indicates that drought stress suppresses the expression of core photosynthetic genes—including *cab*, *psbP*, *psbA*, *psbD*, *petA*, and *petB*—across maize genotypes. This repression is consistently more severe in drought–sensitive varieties than in their drought–tolerant counterparts [14, 22]. This study aimed to (1) compare the physiological and molecular responses to exogenous melatonin in drought–sensitive and drought–tolerant maize genotypes, (2) clarify the underlying mechanisms of melatonin–induced drought tolerance, and (3) assess the practical potential of melatonin application for enhancing crop resilience under field conditions.

## Materials and methods

**Fig. 1:**
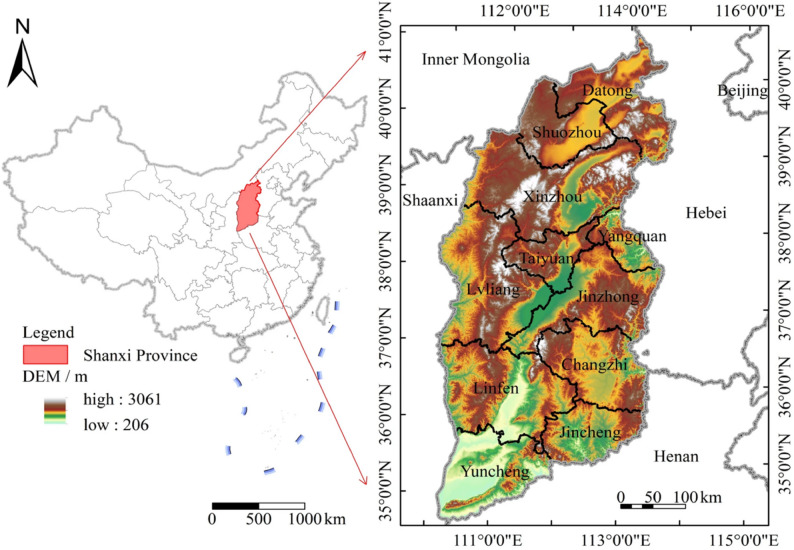
Location of Shanxi Province. Maps were created using ArcGIS10.2 (Environmental Systems Research Institute, USA. https://www.esri.com/)

A field trial was conducted during the 2021–2022 maize growing season at the experimental station of Shanxi Agricultural University in Taigu, Shanxi Province (34° 35′ N, 110° 15′ E) (Fig. 1). The region has a semi–arid, warm temperate continental climate with an average annual precipitation of 491 mm, approximately 60–70% of which falls between July and September. The study compared two maize hybrids: drought–resistant Shaishuang–609 and drought–sensitive Shaishuang–902.

### Experimental design and treatments

The research design was single–factor randomized block cultivar “ Shaishuang–609 (with strong drought resistance) and Shaishuang–902 (with weak drought resistance)” were attained from Taigu Agriculture Bureau, China. Healthy and uniform maize seeds (*Zea mays* L.) were sterilized in a 0.3% sodium hypochlorite (NaClO) solution for 10 min, rinsed thoroughly three times with sterile distilled water, air–dried, and stored at 4 °C until use. In plastic buckets (inner diameter of 26 cm and depth of 38 cm) with the same specifications, 18 kg of air–dried soil was placed in each bucket, containing 1.62% organic matter and 0.064% total nitrogen. Before the drought stress treatment, there were three uniformly grown plants in each bucket, and the soil moisture content was maintained at 80% of the field capacity. At the seedling stage (six leaves), the maize plants were subjected to drought treatment, and 100 µmol·L^–1^ melatonin was sprayed at dusk when the light was weak, and the spraying volume stopped when the water began to drip from the leaves. Each variety had six treatments: CK, normal water supply (soil moisture content at 80% of the field capacity), and leaf spraying with distilled water; CK + MT: normal water supply (soil moisture content at 80% of the field capacity), and leaf spraying with 100 µmol·L^–1^ melatonin; MS: leaf sprayed with distilled water until moderate drought stress (soil moisture content at 50% of the field capacity); MS + MT: leaf spraying with 100 µmol·L^–1^ melatonin until moderate drought stress; SS: leaf spraying with distilled water until severe drought stress (soil moisture content at 35% of the field capacity); and SS + MT: leaf spraying with 100 µmol·L^–1^ melatonin until severe drought stress. Each treatment had 6 replicates. When the soil moisture content was maintained at 80% (control, CK), 50% (moderate drought stress, MS), and 35% (severe drought stress, SS) for 7 days (Fig. 1S).

### Dry matter accumulation

Measurement of biomass accumulation: Six uniformly growing maize plants from each treatment were randomly selected, blanched at 105 °C for 30 min, dried at 65 °C until constant weight was reached, and the dry weight of the plants was measured using an analytical balance.

### Chlorophyll content (SPAD)

Chlorophyll content was determined non–destructively using a SPAD–502 chlorophyll meter (Konica Minolta, Tokyo, Japan). Measurements were taken from six representative uniformly growing plants per plot were taken from the central portion of the youngest fully expanded leaf, and the mean of three readings per leaf was recorded.

### Photosynthetic gas exchange parameters

The gas exchange parameters of three uniformly growing plants from each treatment in the second unfolded leaf were measured using the LI–6400 photosynthesis measurement system (LI–COR, US) in the morning on a sunny day, including the photosynthetic rate (Pn) in the middle of the leaf, stomatal conductance (Gs), intercellular CO_2_ concentration (Ci), and transpiration rate (Tr). The instrument used red and blue light sources for measurement, the photosynthetically active radiation (PAR) in the leaf chamber was set at 1000 µmol (CO_2_)·m^–2^·s^–1^, and the leaf chamber temperature was maintained at 25 °C.

### Chlorophyll a fluorescence parameters

For each treatment, plants exhibiting uniform growth were selected per replicate. The second fully expanded leaf from each selected plant was used for all subsequent measurements. The rapid kinetic curve (OJIP curve) of chlorophyll fluorescence and related parameters and the 820 nm light reflection curve [23] were measured on the leaves after dark adaptation for 60 min using a plant efficiency instrument (M–PEA, UK). The four parameters were the absorption–based PSII performance index (PIABS), maximum photochemical efficiency of PSII (Fv/Fm), relative fluorescence intensity at 300 µs (Vk), relative fluorescence intensity at 2 ms (Vj), and relative fluorescence intensity at 30 ms (Vi).

### Energy conversion efficiency of photo system

Photosystem I (PSI) and II (PSII) quantum yields were measured on the second fully expanded leaf using a Dual–PAM–100 fluorometer (Heinz Walz, Germany). Following dark adaptation (30 min), light–adapted and maximum fluorescence (Fs, Fm′) were recorded to calculate the effective quantum yield of PSII, Y(II). The maximum P700 signal (Pm) was obtained under a saturating pulse with far–red light, and the intermediate signal (P) was measured under actinic light. Key parameters were derived as follows: non–regulatory energy dissipation Y(NO) = Fs/Fm, regulatory dissipation Y(NPQ) = Fs/Fm′ – Fs/Fm, donor–side limitation Y(ND) = (P – P₀)/(Pm – P₀), and acceptor–side limitation Y(NA) = (Pm – Pm′)/(Pm – P₀). The effective PSI quantum yield was calculated as Y(I) = (Pm′ – P)/(Pm – P₀) [24].

### Enzyme activities related to photosynthesis

The leaf samples (1 g) were ground in 9 g 100 mmol·L^–1^ pre–cooled phosphate buffer (pH 7.2–7.4). The extract was centrifuged at 3,000×g at 4 °C for 20 min, and the supernatant was used for enzyme activity analysis. According to the manufacturer’s instructions, the activities of photosynthetic enzymes such as CA, PEPC, PPDK, NADP–ME, and Rubisco were determined using an ELISA kit (Jing’an, Shanghai). The absorbance (OD value) was measured at 450 nm using an enzyme reader, and the activities of the plant photosynthetic enzymes in the samples were calculated using a standard curve.

### Determination of ascorbic acid (AsA) and glutathione (GSH) contents

Glutathione and ascorbic acid contents were determined, and leaf samples (0.3 g) were quickly placed in an ice–cooled mortar, and 4 mL of 5% (w/v) pre–cooled phosphoric acid was used for extraction. The sample was centrifuged at 4000×g for 30 min at 4 °C, and the supernatant was used for determination. To determine the total ascorbic acid content, 200 µL of the supernatant was successively added to 48 µL of triethanolamine (1.84 mol·L^–1^), 802 µL phosphate buffer solution (pH 7.5, 50 mmol·L^–1^, 2.5 mmol·L^–1^ EDTA), 100 µL dithiothreitol (DTT) (10 mmol ·L^–1^), and kept at 25 °C for 10 min to reduce DHA (dehydroascorbic acid) to AsA. Then, 100 µL N–ethylmaleimide (0.5%, w/v) was added to the reaction mixture to remove excess DTT, followed by 400 µL trichloroacetic acid (10%, w/v), 400 µL phosphoric acid (44%, v/v), 400 µL 2,2’–bipyridine (4% [w/v] dissolved in 70% ethanol), and 200 µL FeCl3 (3%, w/v). The reaction mixture was incubated at 37 °C in a constant–temperature water bath for 60 min. To determine AsA, 200 µL PBS (pH 7.5, 50 mmol·L^–1^, 2.5 mmol·L^–1^ EDTA) was used instead of DTT and N–ethylmaleimide. The absorbance at 525 nm was recorded, and the total AsA and reduced AsA content were calculated using a standard curve. DHA content was calculated by subtracting the reduced AsA content from the total ascorbic acid content.

To determine the total glutathione content, 100 µL of the supernatant was successively added to 100 µL of sulfosalicylic acid (5%, w/v), 48 µL of triethanolamine (1.84 mol·L^–1^), and 100 µL of 70% ethanol. The mixture was incubated at 25 °C for 60 min to mask the derivative GSH. Then, 1412 µL PBS (pH 7.5, 50 mmol·L^–1^, 2.5 mmol·L^–1^ EDTA), 40 µL NADPH (10 mmol·L^–1^), and 160 µL 5,5’–dithiobis(2–nitrophenylacetic acid) (DTNB, 12.5 mmol·L^–1^) were added, and the mixture was maintained at 25 °C for 10 min. Subsequently, 40 µL of glutathione reductase (50 U/mL) was added to initiate the reaction,and the change in absorbance at 412 nm was monitored. For oxidized glutathione (GSSG), 70% ethanol was replaced with 2–ethynylpyridine (10% [w/v]). The total glutathione and GSSG contents were calculated based on a standard curve. The reduced glutathione (GSH) content was calculated by subtracting GSSG from the total glutathione content.

### Determination of H_2_O_2_, MDA and superoxide anion contents

Leaves (0.5 g) from each treatment group were collected and placed in an ice–cooled mortar. Add 1 mL of 0.05 mol·L^–1^ phosphate buffer (pH = 7.8) and ground into a paste on an ice bath. Buffer was added to make a final volume >of 10 mL. The mixture was centrifuge at 4 °C and 4000×g for 20 min. The supernatant was stored in a refrigerator at 4 °C for determination of H_2_O_2_, MDA, and superoxide anion content.

### Hydrogen peroxide (H₂O₂) content

H₂O₂ concentration was determined spectrophotometrically. An aliquot of supernatant (1 mL) was mixed with 1 mL of potassium iodide (1 mol·L⁻¹) and incubated in the dark at 25 °C for 1 h. Absorbance was measured at 390 nm, and H₂O₂ content was quantified using a standard calibration curve.

### Malondialdehyde (MDA) content

MDA content, an indicator of lipid peroxidation, was assayed following the supernatant (1 mL) was combined with 2 mL of thiobarbituric acid (0.6%, w/v), heated in a boiling water bath for 15 min, and cooled. The mixture was centrifuged at 4000 × g for 15 min, and absorbance was recorded at 450, 532, and 600 nm.

### Superoxide anion (O₂·⁻) content

Superoxide anion content was measured by hydroxylamine oxidation. The supernatant (0.5 mL) was mixed with 0.5 mL phosphate buffer and 1 mL hydroxylamine hydrochloride (10 mmol·L⁻¹), followed by incubation in the dark at 25 °C for 1 h. Subsequently, 1 mL of p–aminobenzenesulfonic acid (17 mmol·L⁻¹) and 1 mL α–naphthylamine (7 mmol·L⁻¹) were added, and the mixture was incubated for 20 min at 25 °C. Absorbance was measured at 530 nm, and O₂⁻ content was calculated from a standard curve.

### Superoxide dismutase (SOD), peroxidase (POD), and catalase (CAT) activities

SOD (EC 1.15.1.1) activity was determined by monitoring the inhibition of photochemical reduction of nitrotyrosine (NBT). The reaction system included 50 mmol·L^–1^ phosphate buffer (pH 7.8), 130 mmol·L^–1^ Met, 750 mmol·L^–1^ NBT, 100 µmol·L^–1^ EDTA–Na2, 20 µmol·L^–1^ riboflavin, 0.5 mL distilled water, and 0.1 mL crude extract.

According to some modifications, the POD (EC 1.11.1.7) activity was determined by monitoring the increase in absorbance at 470 nm. The reaction system included 0.1 mol·L^–1^ phosphate buffer (pH 6.0), 0.06% pyrogallol, 1.14% H_2_O_2_, and 0.1 mL crude enzyme solution.

CAT (EC 1.11.1.6) activity was determined by monitoring the decomposition of H₂O₂ at 240 nm (ε = 39.4 M⁻¹ cm⁻¹). The reaction mixture consisted of 0.08 mol·L⁻¹ phosphate buffer (pH 7.0) and 0.02 mol·L⁻¹ H₂O₂. The reaction was initiated by adding 100 µL of crude enzyme extract, and the decrease in absorbance was measured at 25 °C.

### Ascorbate peroxidase (APX), monodehydroascorbate reductase (MDHAR), dehydroascorbate reductase (DHAR), and glutathione reductase (GR) enzyme activities

Crude enzyme extraction: Take 0.3 g) were ground in 4 mL of pre–cooled extraction buffer [containing 50 mmol·L^–1^ KH_2_PO_4_–KOH (pH 7.5), 0.1 mmol·L^–1^ EDTA, 20% (v/v) glycerol, and 2% (w/v) polyvinylpyrrolidone] to form a homogenate. The homogenate was incubated at 4 °C for 10 min, followed by centrifuge at 4000×g for 15 min at 4 °C. The supernatant was used to determine APX, MDHAR, DHAR, and GR enzyme activities.

### Activity of the Ascorbate–Glutathione (AsA–GSH) cycle enzyme

The activity of ascorbate peroxidase (APX, EC 1.11.1.11) was measured by monitoring the decrease in absorbance at 290 nm. The reaction system included 50 mmol·L^–1^ Hepes–KOH (pH 7.6), 0.5 mmol·L^–1^ AsA, and 1 mmol·L^–1^ H_2_O_2_, and 50 µL of crude enzyme. H_2_O_2_was added to initiate the reaction at 25 °C.

GR activity of glutathione reductase (EC 1.6.4.2) was measured at 340 nm. 3 mL of the reaction system included 100 mmol·L^–1^ Tris–HCl (pH 8.0), 1 mmol·L^–1^ GSSG, 0.2 mmol·L^–1^ NADPH, and 0.1 mL of crude enzyme, initiated by NADPH.

The activity of dehydroascorbate reductase DHAR (EC 1.8.5.1) activity. The reaction system includes 100 mmol·L^–1^ Hepes–KOH (pH 7.0), 2.5 mmol·L^–1^ GSH, 0.2 mmol·L^–1^ dehydroascorbate salt (DHA), and 100 µL crude enzyme solution. The reaction was initiated with docosahexaenoic acid (DHA) at 25 °C. The activity of a single dehydroascorbate reductase (MDHAR, EC, 1.6.5.4) was measured at 340 nm. The reaction system includes 0.1 mmol·L^–1^ NADH, 0.25 mmol·L^–1^ AsA, 0.3 units ascorbic acid oxidase (AO), and 100 µL crude enzyme. The reaction was initiated by AO at 25 °C.

### Gene expression levels

Total RNA was extracted from maize seedlings, freeze–dried leaf samples (300 mg) were ground into powder in liquid nitrogen, and total RNA was extracted using an RNA extraction kit (TIANGEN, China). Next, 20 μL of the system was reverse–transcribed according to the manufacturer's instructions (FastQuant RT Kit, TIANGEN, China). Specific primers for the genes were designed using the Primer Premier 5.0. According to the manufacturer's instructions (SuperReal PreMix Plus (SYBR Green, TIANGEN, China), 20 μL of real–time fluorescence quantitative PCR (qRT–PCR) was conducted using the BIO–RAD CFX96 instrument. The reaction was performed in a three–step protocol. The reaction conditions were as follows: 15 min of pre–denaturation at 95°C and 40 cycles of 10 s at 95°C, 20 s at 55°C, and 20 s at 72°C. GADPH (gene ID: 542367) was used as an internal reference for normalization of gene expression. The expression levels of antioxidant enzyme genes such as SOD, POD, CAT, APX, MDHAR, DHAR, and GR in different drought–resistant maize leaves, as well as the changes in the expression levels of photosynthesis–related genes PEPC, PPDK, NADP–ME, psaA, psaB, petA, petB, psbA, psbB, psbC, psbD, and psbP were determined (Table 1).

**Table 1:** The specific primer of genes

Gene Name	Gene ID	Sense Primer	Anti–sense primer
*GAPDH*	542,367	CCATCACTGCCACACAGAAAAC	AGGAACACGGAAGGACATACCAG
*APX2*	542,476	CAACGCCGGACTGGAAATTG	TCAGAACCTTGGGTGGCATC
*DHAR1*	100,273,125	CAAAGCTCATGGCCCCTACA	GCTCCTCGGATGGCTTAGTC
*GR1*	541,986	TTGGCATTCACCCCACATCC	ATACTCCCTCCCCCGAGAAC
*MDHAR1*	100,501,585	GAGTTAAGCCTGGGGAGCTT	AGGCAATAGCTTCTCTCCACC
*CAT1*	542,369	TGGAACAACAACTCTGCCCT	GCACGGAGAAAATCAGCACA
*POX1*	542,029	TCGCGTGGACTGTGGTTTT	ATAGTCCTAAGCCAGCAGCA
*SOD4*	542,722	TCAGGAAGAGCACCGGAAAC	GATGATCCCTGTGGTGGCAA
*CA*	100,280,638	CAGGGACATCAGCATCTT	AAACAAAACCGTAACCATA
*PEPC*	542,372	GAAGACACGCTCATCCTCACC	CAGTTCGGCATTTCCATCC
*PPDK*	542,759	GAAGGTGGGCATTTGTGG	AAGGGAGATGGGAGTGTAGC
*NADP–ME*	542,233	CCCAGCGACCTGGTGAAA	TCCAGCAGCACCAGCAAC
*rbcL*	845,212	GTGTCTACGCGGTGGACTTG	TGATTTCACCAGTTTCGGCT
*rbcS*	542,212	AACGGCTGGATACCCTGCCTCGAGT	CTGCGTCTGCTTGATGTTGTCG
*psbA*	845,199	TCGCTGCTCCTCCAGTAGAT	CGCATACCCAGACGGAAACT
*psbB*	845,200	AGGCTCAATGGACAATGGGG	CCACCGTTACGCCTACTTGT
*psbC*	845,201	CTACCACGTGGAAACGCTCT	TCTGGTACGAAATGGGCCAC
*psbD*	845,202	TCTGGTTGGTTCTTCTCGCC	AGGTATTTGCACCATCGCCA
*psbP*	100,281,199	GGTTGCCGAGGCTCTGATTA	TCTCGTTTGCTCCAGTGGTC
*petA*	845,191	GAAACCGAGGAAGGGGACAG	TTCTGGCCCGGGAGGTATAA
*petB*	845,192	TATACGGTTCTCGGAGGGGG	AAAGCCTCTGTAACGGTCGG
*psaA*	845,195	TATTTGCTCGCAGTTCCCGT	TACCCCAAACATCCGACTGC
*psaB*	845,196	GCAGGGCAACGTTTCACAAT	GCCAATATCCACGCCAGGAA

### Data analysis

The experimental data was collected and collated in Excel 2010 (Microsoft Corp., USA), Figures were created using the Origin 8.0 software (Origin Lab Corp., MA, USA). For dry matter translocation before anthesis and dry matter accumulationafter the anthesis of winter wh, yield and its components, a two–way analysis of variance (ANOVA) was performed using SPSS, version 19.0 (SPSS Inc., USA). The mean values of all treatment were compared for any significant differences using the Duncan’s multiple range tests at a significance level of *P* < 0.05 using the SPSS 19.0.The cluster heatmap and correlation matrix plot were generated based on the relationship among water consumption, dry matter accumulation, and yield components of maize using the R packages “pheatmap” and “corrplot”, respectively.

## Results

### Exogenous melatonin enhances leaf chlorophyll content and dry matter accumulation in drought–stressed maize

Different drought resistance–type maize growth under the influence of exogenous melatonin on drought stress of maize leaf chlorophyll content (SPAD), the influence of the amount of dry matter accumulation can be seen in (Table 1S). Under normal water supply conditions, The SPAD values and dry matter accumulation of SD–609 and SD–902 leaves were not affected by exogenous melatonin treatment. Under moderate and severe drought stress, the SPAD values and dry matter accumulation in the leaves of SD–609 and SD–902 plants decreased significantly. However, the SPAD values and dry matter accumulation of plants treated with melatonin were higher than those of untreated plants. 

### Exogenous melatonin improves leaf gas exchange in drought–stressed maize

**Fig. 2:**
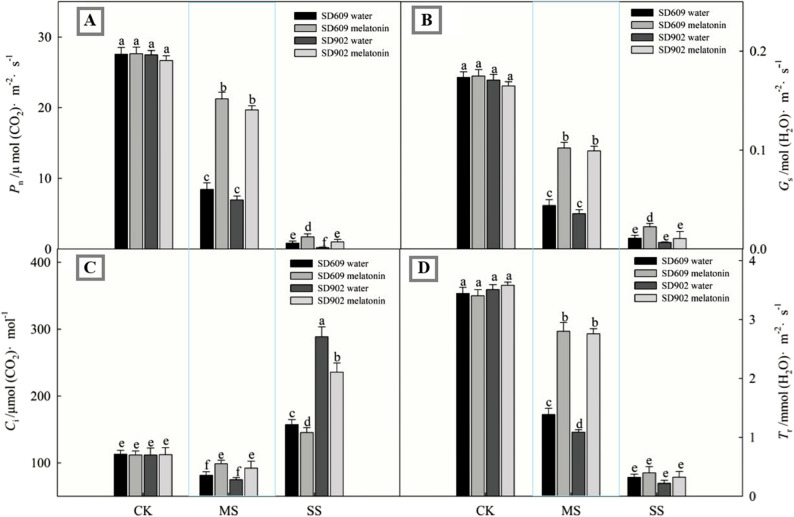
Gas exchange responses of SD–609 and SD–902 maize varieties to exogenous melatonin under drought stress. Note: (**A**); Pn: photosynthetic rate; (**B**); Gs: stomatal conductance; (**C**); Ci: intercellular CO2 concentration; (**D**); Tr: transpiration rate

The photosynthetic rate under drought stress can directly reflect the growth status of plants. As shown in (Fig. 2), under moderate and severe drought stress conditions, compared with normal water supply treatment, the net photosynthetic rate (Pn), stomatal conductance (Gs), and transpiration rate (Tr) of the leaves of SD–609 and SD–902 all showed a significant downward trend. Under moderate and severe drought stress, the intercellular CO_2_ concentration (Ci) in the leaves of SD–609 and SD–902 showed a trend of first decreasing and then increasing. In addition, under moderate and severe drought stress, the net photosynthetic rate and stomatal conductance of SD–609 leaves were higher than those of SD–902. Under moderate and severe drought stress conditions, exogenous melatonin treatment inhibited the decline in photosynthetic rates of SD–609 and SD–902 caused by drought stress, increased stomatal conductance and transpiration rate, regulated intercellular CO_2_ concentration, and improved the growth status.

### Effects of exogenous melatonin on the OJIP curve of maize under drought stress

The JIP test can be used to quantitatively analyze the parameters related to the OJIP curve and reflect the functional and structural changes in the photosynthetic system under environmental stress conditions. As shown in (Table 2), drought stress significantly reduced the photosynthetic performance index PIABS and the maximum photochemical efficiency Fv/Fm of PSII in SD–609 and SD–902. Moreover, compared to SD–609 and SD–902 had higher PIABS and Fv/Fm values under drought stress. Compared with the plants that did not receive melatonin treatment, the plants treated with melatonin in SD–609 and SD–902 under drought stress conditions had higher PIABS and Fv/Fm and decreased Vj, Vk, and Vi.

**Table 2:** Effects of exogenous melatonin on chlorophyll a fluorescence parameters of drought–tolerant (SD–609) and drought–sensitive (SD–902) maize genotypes under drought stress

Variety	Parameter	PIABS	F_v_/F_m_	V_j_	V_k_	V_i_
SD–609	CK	2.258 ± 0.042a	0.821 ± 0.003a	0.499 ± 0.004d	0.251 ± 0.002c	0.805 ± 0.004b
	CK + MT	2.242 ± 0.029a	0.820 ± 0.002a	0.496 ± 0.004d	0.251 ± 0.002c	0.803 ± 0.005b
	MS	1.518 ± 0.061c	0.807 ± 0.003b	0.552 ± 0.006b	0.302 ± 0.004b	0.826 ± 0.005ab
	MS + MT	1.726 ± 0.062b	0.815 ± 0.003a	0.533 ± 0.007c	0.294 ± 0.005b	0.813 ± 0.007b
	SS	1.236 ± 0.063d	0.793 ± 0.004c	0.577 ± 0.007a	0.325 ± 0.008a	0.838 ± 0.011a
	SS + MT	1.557 ± 0.049c	0.803 ± 0.003b	0.541 ± 0.005b	0.294 ± 0.005b	0.822 ± 0.009ab
SD–902	CK	2.166 ± 0.035a	0.815 ± 0.003a	0.503 ± 0.002d	0.248 ± 0.002d	0.809 ± 0.002b
	CK + MT	2.249 ± 0.031a	0.818 ± 0.002a	0.499 ± 0.003d	0.246 ± 0.003d	0.809 ± 0.005b
	MS	1.278 ± 0.049c	0.803 ± 0.003b	0.568 ± 0.006b	0.337 ± 0.005b	0.838 ± 0.003ab
	MS + MT	1.598 ± 0.067b	0.808 ± 0.003a	0.549 ± 0.007c	0.295 ± 0.005c	0.819 ± 0.003b
	SS	1.007 ± 0.083d	0.787 ± 0.008c	0.622 ± 0.014a	0.365 ± 0.013a	0.857 ± 0.016a
	SS + MT	1.382 ± 0.081c	0.811 ± 0.003a	0.580 ± 0.010b	0.336 ± 0.011b	0.834 ± 0.005ab

PI_ABS_: PSII performance index on an absorption basis; F_v_/F_m_: the maximal PSII photochemistry efficiency; V_k_: the relative fluorescence intensity at 300 µs; V_j_: the relative fluorescence intensity of the J–step (2 ms); V_i_: the relative fluorescence intensity of the I–step (30 ms)

### Effects of exogenous melatonin on the PSII characteristics and electron transfer of maize under drought stress

**Fig. 3:**
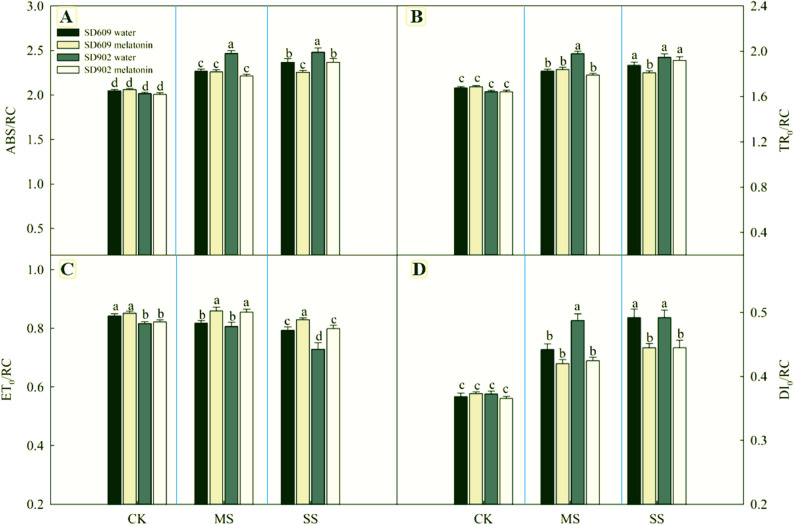
Effects of exogenous melatonin on PSII reaction center of SD–609 and SD–902 under drought stress Note: **A** Average absorbed photon flux per active PSII [ABS/RC]. Larger light–harvesting complexes fewer active RCs (e.g., photoinactivation), ABS/RC = (M₀/Vⱼ)(1/φP₀); (**B**) Maximum trapped exciton flux per active PSII [TR_0_/RC], Impaired charge separation inactive oxygen–evolving complex Formula: TR₀/RC = M₀/Vⱼ (**C**) The flux of energy dissipated in processes other than trapping per active PSII [DI_0_/RC], High–light stress thermal dissipation (NPQ) activation DI₀/RC = ABS/RC – TR₀/RC (**D**): The flux of electrons transferred from QA– to PQ per active PSII [ET_0_/RC], QB–site inhibition (herbicides) Limited plastoquinone pool oxidation ET₀/RC = (M₀/Vⱼ)ψ₀

Under conditions of moderate and severe drought stress, SD–609 and SD–902 units PSII reaction center (RC) absorbed energy (ABS/RC), capture energy (TR0 / RC), and the energy for heat dissipation (DI0/RC) increased significantly with the intensification of drought stress, while the quantum yield per reaction center for electron transfer (ET0/RC) decreased significantly (Fig. 3), indicating that drought stress led to the inactivation of some PSII reaction centers in SD–609 and SD–902. This increases the burden on the remaining active reaction centers and reduces the electron transfer efficiency. Exogenous melatonin significantly alleviated the excitation energy stress of PSII response centers in SD–609 and SD–902 under drought stress, reduced ABS/RC, TR0/RC, and DI0/RC, promoted photosynthetic electron transfer under drought stress, and enhanced PSII activity. Electrons from QA passed to the quantum yield of PQ (E0), reaction center to capture the exciton transfer electrons from QA to the efficiency of the PQ (0), electronic transmission between from the system to the probability of PSI receptor side electron acceptor (R0). The quantum yield of PSI terminal receptor reduction (R0) was mainly used to reflect the changes in photosynthetic electron transfer in plants under stress conditions. Drought stress not only inhibits PSII activity but also hinders normal photosynthetic electron transfer. As shown in (Table 3), moderate and severe drought stress significantly reduced the 0, E0, and R0 of SD–609 and SD–902 and had a relatively small effect on R0. Exogenous melatonin reversed the adverse effects of drought stress on photosynthetic electron transport, restoring various indicators to a certain extent and promoting the activity of the electron transport chain under drought stress conditions.

**Table 3:** Effects of exogenous melatonin on the photosynthetic electron transport of SD–609 and SD–902 under drought stress

Variety	Parameter	ϕE_0_	Ψ_0_	ϕR_0_	δR_0_
SD–609	CK	0.413 ± 0.003a	0.503 ± 0.004a	0.163 ± 0.004a	0.394 ± 0.008a
	CK + MT	0.414 ± 0.003a	0.506 ± 0.004a	0.163 ± 0.005a	0.392 ± 0.009a
	MS	0.362 ± 0.006c	0.450 ± 0.006c	0.142 ± 0.004b	0.390 ± 0.008a
	MS + MT	0.382 ± 0.006b	0.469 ± 0.008b	0.154 ± 0.003a	0.404 ± 0.009a
	SS	0.338 ± 0.007d	0.425 ± 0.008d	0.129 ± 0.005c	0.385 ± 0.004a
	SS + MT	0.369 ± 0.005c	0.460 ± 0.006b	0.144 ± 0.004b	0.390 ± 0.009a
SD–902	CK	0.406 ± 0.003a	0.498 ± 0.003a	0.157 ± 0.002a	0.385 ± 0.004ab
	CK + MT	0.409 ± 0.003a	0.502 ± 0.004a	0.158 ± 0.005a	0.385 ± 0.009ab
	MS	0.348 ± 0.006c	0.433 ± 0.007c	0.132 ± 0.003b	0.378 ± 0.008b
	MS + MT	0.366 ± 0.007b	0.452 ± 0.008b	0.147 ± 0.003a	0.402 ± 0.009a
	SS	0.299 ± 0.013d	0.379 ± 0.015d	0.114 ± 0.002c	0.381 ± 0.008ab
	SS + MT	0.342 ± 0.009c	0.421 ± 0.011c	0.137 ± 0.006b	0.398 ± 0.011a

E_0_: Quantum yield of electron transport from Q_A_^–^ to PQ; _0_: Efficiency with which a PSII trapped electron is transferred from Q_A_^–^ to PQ; R_0_: Quantum yield of electron transport from Q_A_^–^ to final PSI acceptors; R_0_: Probability that an electron is transported from the reduced intersystem electron acceptors to the final electron acceptors of PSI

### Effects of exogenous melatonin on the 820 nm light reflection curve (MR/MR0) and PSI characteristics of maize under drought stress

The MR/MR0 curve consists of decreasing and increasing phases. The decreasing phase reflected the oxidation rates of PSI and PC, whereas the increasing phase reflected the reduction rates of PSI and PC. The lowest point of MR/MR0, the PC and PSI reduction rates were equal to their oxidation rates. As shown in (Fig. 2S), under drought stress conditions, the MR/MR0 values of SD–609 and SD–902 significantly changed. The lowest point of MR/MR0 increased with increasing drought stress intensity and melatonin treatment alleviated the influence of drought stress on the MR/MR0 curve. To quantitatively analyze the changes in the oxidation and reduction rates of PSI and PC in maize under drought stress, several PSI parameters were obtained from MR/MR0 (Table 4). Under moderate and severe drought stress, the oxidation rates of PSI and PC in SD–609 and SD–902 (VPSI) significantly decreased, and the reduction rates of PSI and PC as reflected by VPSII–PSI also significantly decreased under severe drought stress. Compared with untreated plants under drought stress, SD–609 and SD–902 plants treated with melatonin had higher VPSI and VPSII–PSI PSI, and the PC of the plants treated with melatonin maintained relatively high oxidative and reducing activities.

**Table 4:** Effects of exogenous melatonin on the parameters of MR/MR0 of SD–609 and SD–902 under drought stress

Variety	Parameter	VPSI	VPSII–PSI	VPSII
SD–609	CK	2.017 ± 0.023a	0.190 ± 0.014a	2.206 ± 0.034a
	CK + MT	2.064 ± 0.013a	0.195 ± 0.014a	2.258 ± 0.025a
	MS	1.813 ± 0.016b	0.188 ± 0.019a	1.999 ± 0.034b
	MS + MT	2.020 ± 0.020a	0.193 ± 0.018a	2.212 ± 0.037a
	SS	1.521 ± 0.027d	0.157 ± 0.017b	1.677 ± 0.043d
	SS + MT	1.620 ± 0.045c	0.184 ± 0.013a	1.803 ± 0.054c
SD–902	CK	1.940 ± 0.033a	0.175 ± 0.013a	2.113 ± 0.042a
	CK + MT	2.001 ± 0.036a	0.171 ± 0.024a	2.171 ± 0.055a
	MS	1.740 ± 0.025b	0.161 ± 0.017a	1.899 ± 0.043b
	MS + MT	1.916 ± 0.037a	0.167 ± 0.018a	2.081 ± 0.053a
	SS	1.421 ± 0.043c	0.149 ± 0.012b	1.569 ± 0.052c
	SS + MT	1.412 ± 0.029c	0.163 ± 0.011a	1.574 ± 0.039c

V_PSI_: Maximum decreasing rate of MR/MR_0_, which represents the oxidation rate of PSI and PC; V_PSII–PSI_: Maximum increasing rate of MR/MR_0_, which represents the reduction rate of PSI and PC

### Effects of exogenous melatonin on the activity and gene expression of antioxidant enzymes in maize under drought stress

**Fig. 4:**
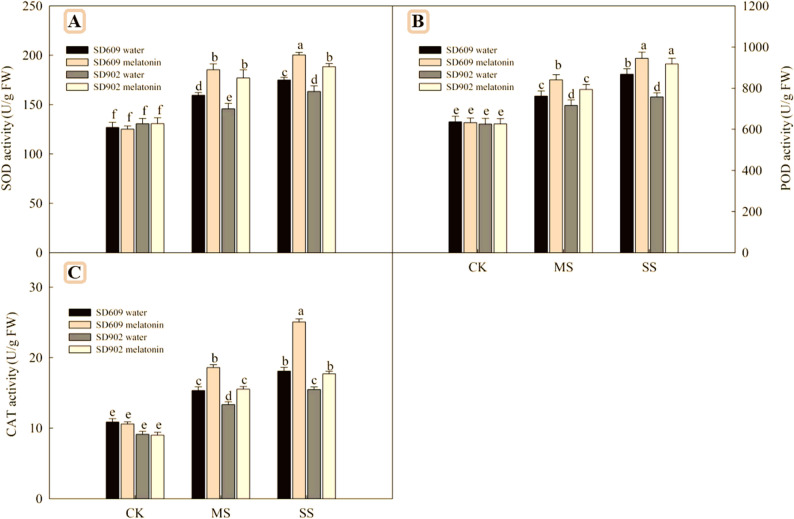
Effects of exogenous melatonin on the activity of antioxidant enzymes of SD–609 and SD–902 under drought stressNote: (**A**): the activity of superoxide dismutase (SOD); (**B**): the activity of peroxidase (POD); (**C**): the activity of catalase (CAT)

SOD, POD, and CAT are important antioxidant enzymes in plants. As shown in (Fig. 4), under moderate and severe drought stress conditions, the enzyme activities of SOD, POD, and CAT in SD–609 and SD–902 increased with the intensification of drought stress, and the enzyme activities of SOD, POD, and CAT in SD–609 were higher than those in SD–902. Additionally, the enzyme activities of APX, MDHAR, GR, and DHAR, involved in the ASA–GSH cycle in.

**Fig. 5:**
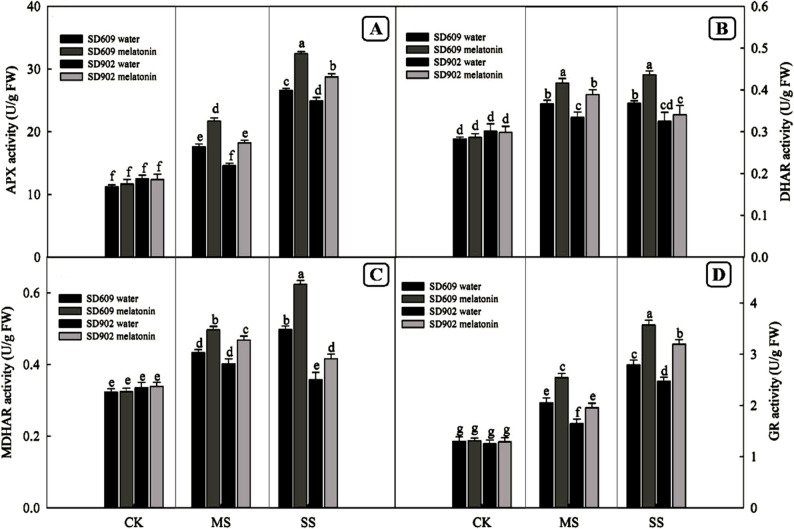
Effects of exogenous melatonin on the activity of AsA–GSH cycle–related enzymes of SD–609 and SD–902 under drought stress. Note: (**A**): the activity of ascorbate peroxidase (APX); (**B**): the activity of dehydroascorbate reductase (DHAR); (**C**): the activity of monodehydroascorbate reductase (MDHAR); (**D**):the activity of glutathione reductase (GR)

**Fig. 6:**
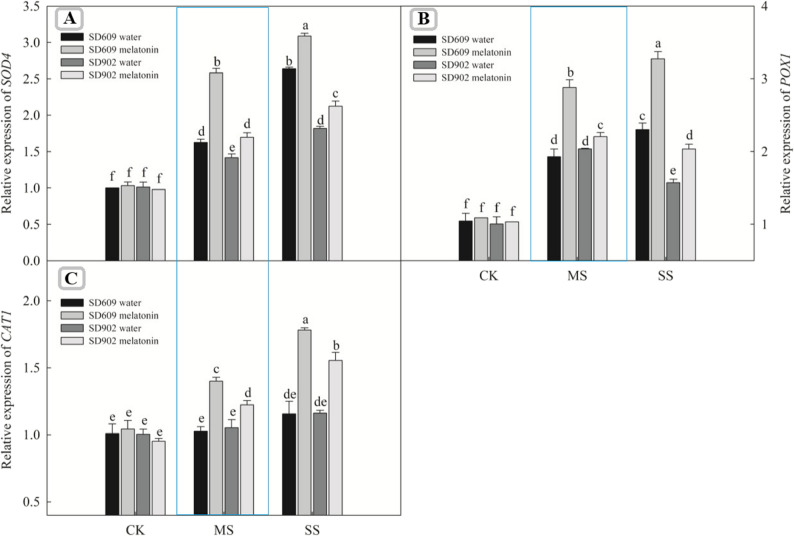
Effects of exogenous melatonin on the genes relative expression of SOD4, POX1 and CAT1 of SD–609 and SD–902 under drought stressNote: (**A**): the relative expression of SOD4 gene; (**B**): the relative expression of POX1 gene; (**C**): the relative expression of CAT1 gene

**Fig. 7:**
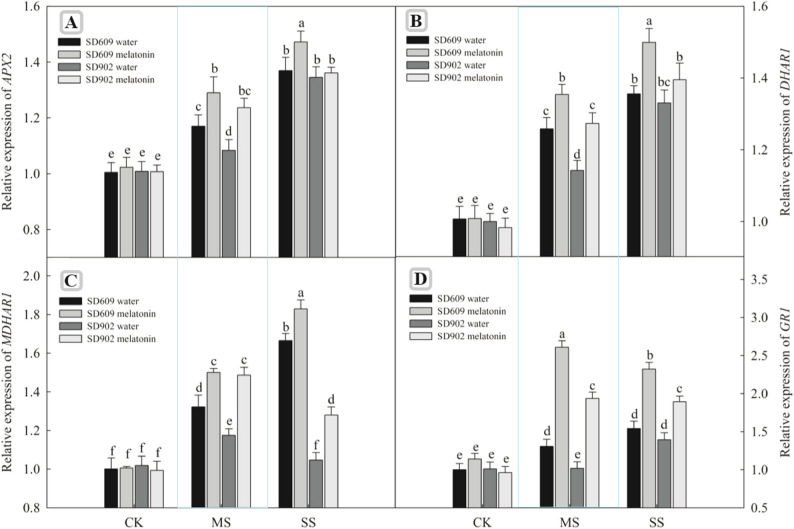
Effects of exogenous melatonin on the genes relative expression of *APX2*, *DHAR1*,* MDHAR1* and *GR1* of SD–609 and SD–902 under drought stressNote: (**A**): the relative expression of APX2 gene; (**B**): the relative expression of *DHAR1* gene; (**C**): the relative expression of *MDHAR1*gene; (**D**): the relative expression of *GR1* gene

SD–609 and SD–902 also increased under moderate and severe drought stress conditions (Fig. 5). Meanwhile, exogenous melatonin treatment further enhanced the activities of these seven enzymes in SD–609 and SD–902 under drought stress. Quantitative real–time PCR revealed that the transcriptional levels of genes encoding antioxidant enzymes (SOD4, POX1, CAT1, APX2, MDHAR1, GR1, and DHAR1) in the leaves of maize seedlings were affected by drought stress and melatonin treatments (Figs. 7 and 6). Under moderate drought stress, the relative expression levels of SOD4, POX1, CAT1, APX2, MDHAR1, GR1, and DHAR1 in SD–609 and SD–902 treated with melatonin were significantly increased. Under severe drought stress, melatonin significantly upregulated the expressionof SOD4, POX1, CAT1, MDHAR1, GR1, and other genes in SD–609, while in SD–902, melatonin treatment only upregulated SOD4, POX1, CAT1, MDHAR1, and GR1.

### Correlation matrix of maize leaf morphological and physiological indicators under drought and melatonin treatments

**Fig. 8:**
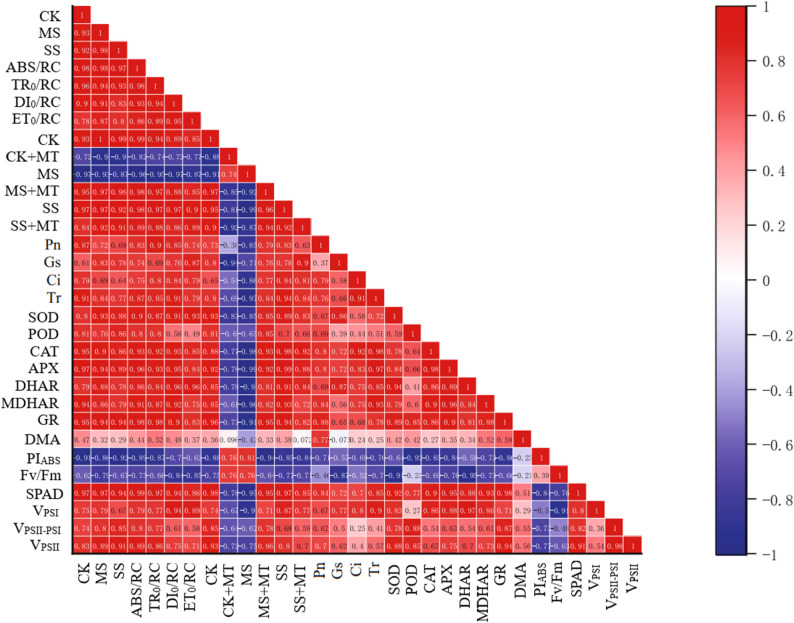
Correlation matrix between leaf morphological and physiological indicators of maize

This correlation matrix effectively illustrates the complex interplay between leaf physiology, biochemistry, and ultimate growth in maize, highlighting how melatonin’s mitigating effect likely operates by strengthening the positive correlations within the antioxidant and photosynthetic systems while preventing the negative shift in photosystem balance under drought stress (Fig. 8). Photosynthesis & Gas Exchange: Pn (Net Photosynthesis) will be strongly positively correlated with Gs (Stomatal Conductance) and Fv/Fm (PSII health). Antioxidant Defense: The antioxidant enzymes (SOD, POD, CAT, APX) will likely show positive correlations with each other, indicating a coordinated response to stress. Growth & Photosynthesis: DMA (Dry Matter Accumulation) and SPAD (Chlorophyll) will positively correlate with high Pn. Drought treatments (MS, SS) will be negatively correlated with Pn, Gs, Fv/Fm, and DMA. The RC/ET ratio (indicating damage) will be negatively correlated with Fv/Fm and ΦPSII.

## Discussion

Maize is one of the main food crops grown in the northwestern region. Drought–induced yield reduction accounts for approximately 15% of the potential maize yield. Photosynthesis is a crucial metabolic process for maize growth and development. Inhibition of the photosynthetic process ultimately affects the yield. Moreover, the response of maize to drought stress is not only closely related to the genotype, but also depends on the severity and duration of stress [24]. Currently, external application of plant growth regulators (such as osmotic protectants, antioxidants, and growth promoters) is considered an effective method to enhance the drought resistance of plants in crop production. Melatonin, a powerful antioxidant and plant growth regulator, plays an important role in enhancing plant stress resistance [25]. Therefore, exploring the physiological mechanisms by which melatonin regulates the drought resistance of different drought–tolerant maize varieties, especially clarifying the response mechanisms of the photosynthetic process and antioxidant system of different drought–tolerant maize varieties to exogenous melatonin, can provide a theoretical basis and technical support for the application of melatonin in improving maize drought resistance and cultivation regulation.

### Effects of exogenous melatonin on the growth of different drought–tolerant maize plants under drought stress

Photosynthesis is a key plant metabolic process. Drought stress first causes the closure of plant stomata to reduce transpiration, hindering the entry of CO_2_ into mesophyll cells and affecting the photosynthesis of maize leaves through stomatal limitation factors and non–stomatal limitation factors. This study showed that under moderate drought stress conditions, the net photosynthetic rate (Pn), stomatal conductance (Gs), and intercellular carbon dioxide concentration (Ci) of SD–609 and SD–902 leaves significantly decreased, indicating that stomatal limitation was the main reason for the decrease in photosynthetic rate. Under severe drought stress conditions, the Pn and Gs of leaves showed a downward trend, whereas Ci increased, indicating that non–stomatal limitation is the main reason for the decrease in the photosynthetic rate of maize leaves under severe drought stress [25, 26]. Exogenous melatonin increased the net photosynthetic rate and stomatal conductance of SD–609 and SD–902 maize plants under drought stress and regulated the intercellular carbon dioxide concentration, indicating that exogenous melatonin may participate in the regulation of stomatal opening and the alleviation of non–stomatal limitation factors, thereby promoting the photosynthetic capacity of maize plants. In addition, exogenous melatonin inhibited the decrease in chlorophyll content under drought stress,possibly because of the downregulation of chlorophyll degradation genes under stress conditions [27]. Melatonin improved the photosynthetic characteristics of SD–609 and SD–902 plants under drought stress, thus promoting plant growth and increasing dry matter accumulation. It can more sensitively respond to environmental stress to reflect the overall photosynthetic capacity of PSII, density of the open PSII reaction center, quantum yield of the primary photochemical reaction, and electron transfer efficiency [28]. In this study, the higher PI_ABS_ and Fv/Fm in melatonin–treated SD–609 and SD–902 plants confirmed that melatonin treatment alleviated PSII photoinhibition induced by drought stress. The increase in ABS/RC and TR_0_/RC indicated that drought stress led to the degradation or inactivation of some PSII reaction centers in SD–609 and SD–902, thereby placing open PSII reaction centers under the stress of excessive excitation energy. Moreover, the decrease in ET_0_/RC caused by drought stress indicated that the light energy utilization efficiency of the open PSII reaction centers in SD–609 and SD–902 decreased, which might be due to the blockage of the photosynthetic electron transfer chain and/or the increase in DI_0_/RC. External melatonin treatment restored indicators such as ABS/RC, ET_0_/RC, TR_0_/RC, and DI_0_/RC in SD–609 and SD–902 to a certain extent, indicating that external melatonin improved the activity of PSII reaction centers and photosynthetic electron transfer [29]. This study shows that melatonin treatment alleviated the damage of drought stress to PSII reaction centers, alleviated the influence of excessive excitation energy on PSII reaction centers, actively regulated the energy absorption, capture and electron transfer in PSII, and the higher PSII activity may be related to the improvement of the rapid synthesis and repair of PSII proteins under drought stress by melatonin. The MR signal reflects the changes in the redox states of PC and P700. The decreasing and increasing stages of MR/MR_0_ reflect the oxidative and reductive activities of PSI and PC, respectively [30]. Under drought stress, the MR/MR_0_ curves of SD–609 and SD–902 were significantly different from those of CK, with the lowest increasing point. In melatonin–treated SD–609 and SD–902 plants, the increase in VPSI and VPSII–PSI indicated that melatonin improved the oxidative and reductive activities of PSI under stressful conditions. The improvementin PSI oxidative activity may be attributed to recovery of the PSI receptor. The recovery of PSI reductive activity in melatonin–treated plants may be due to the increase in PSII activity by melatonin treatment, which promotes electron transfer between the light systems and/or the electron transfer chain between PSII and PSI. This study demonstrated that drought stress damages multiple components of the electron transfer chain and disrupts the balance of electron transfer, while exogenous melatonin application can alleviate this phenomenon. The increase in SD–609 and SD–902 Vj caused by drought stress indicates an excessive reduction of QA, reflecting the obstruction of electron transfer from the PSII acceptor side QA to PQH_2_ [31]. At the same time, the increase in SD–609 and SD–902 Vi suggests that drought stress significantly reduces the ability of plastid quinone (PQ) to accept electrons. Additionally, the increase in Vk indicates that the electron transfer capacity of the PSII oxygen–evolving complex (OEC) and the donor side of PSII are affected by drought stress [32]. In this study, the decrease in Vj, Vi, and Vk in melatonin–treated plants indicates that melatonin application improves the redox state of QA and plastid quinone and increases the activity of OEC. In melatonin–treated plants, the electron transfer quantum yield from P680 to QA (Fv/Fm), from QA to plastid quinone (E_0_, 0), from plastid quinone to the PSII terminal acceptor (R_0_), and electron transfer from PSII to PSI increased. The probability of transfer to the PSI receptor side (R_0_) improved, indicating that melatonin improved SD–609 and SD–902 under drought stress. Activity of the entire electron transfer chain from the PSII donor side to PSI terminal receptor side.

### Effects of exogenous melatonin on the light energy conversion efficiency of different drought–resistant types of maize under drought stress

The balance between light energy capture and energy distribution affects photosystem stability. Drought stress can damage photosystem II (PSII), the photochemical efficiency of photosystem I (PSI), inhibit photosynthetic electron transfer, and reduce the fluidity of thylakoid membranes, Rubisco activity, and cell membrane stability [33]. Determination of energy conversion efficiency in PSI and PSII provides comprehensive information on the activity of PSII and PSI under drought stress. Studies have shown that drought stress damages t, thereby reducing the efficiency and electron transfer capacity of PSI and PSII. This study found that under moderate and severe drought stress conditions, Y(NPQ) increased in SD–609 and SD–902 plants, indicating that the PSII reaction center was under excitation energy stress induced by drought stress, and the plants dissipated excess excitation energy through a regulatory non–photochemical energy dissipation mechanism [34]. However, Y(II) in SD–609 and SD–902 treated with melatonin significantly increased, indicating that exogenous melatonin reversed the negative impact of drought stress on the photochemical efficiency of PSII and reduced the passive energy dissipation of maize leaves caused by drought stress, and that SD–609 treated with melatonin under severe drought stress conditions still had a relatively high level of Y(NPQ), which may be an important protective mechanism for SD–609 to alleviate the photoinhibition of PSII. The photoinhibition of PSI is more dangerous than that of PSII because the photoinhibition of PSI is difficult to recover and may also lead to secondary damage [35]. Compared with SD–609, the values of Y(NO) and Y(NA) in SD–902 remained relatively high under drought stress, indicating that the photosynthetic apparatus of SD–902 was more sensitive to drought stress than SD–609. Exogenous melatonin improves the photochemical efficiency of PSI by alleviating the limitations of the donor and acceptor sides and maintaining the stability of the PSI complex. The increase in ETR(II) and ETR(I) in melatonin–treated plants also proves that melatonin treatment reduces the negative impact of drought stress on the photosynthetic apparatus and promotes smooth flow of the electron transfer chain. Therefore, melatonin application is an effective way to promote the utilization of light energy, alleviate PSII and PSI damage induced by drought, and enhance the drought resistance of the photosynthetic apparatus of SD–609 and SD–902. The improvement in photochemical efficiency, energy dissipation, and the activity of the photosynthetic electron transfer chain may be important regulatory strategies of melatonin in helping plants reduce ROS accumulation and adapt to drought stress.

### Effects of exogenous melatonin on the expression characteristics of photosynthetic genes in different drought–tolerant maize varieties under drought stress

The process by which plants respond to drought stress involves not only a series of physiological and biochemical reactions but also changes at the genetic expression level [36]. PSI and PSII are multi–subunit pigment–protein complexes composed of proteins encoded by the nuclear and chloroplast genes, respectively. D1 and D2 proteins, which are the most important components of PSII, form the basic framework of the PSII reaction center. PsaA and PsaB are the protein components of the PSI reaction center. In this study, the expression levels ofpsbA, psbB, psbC, psbD, and psbPgenes in SD–609 and SD–902 PSII and the psaA and psaB genes in PSI were inhibited under drought stress. This reduction may be one of the main reasons for the photoinhibition of PSI and PSII [37]. In this study, exogenous melatonin protected PSII by upregulating the expression levels of psbA, psbB, psbC, psbD,and psbPgenes in the SD–609 and SD–902 PSII under drought stress, explaining the intrinsic reason for the improvement in PSII activity and maximum photochemical efficiency. Simultaneously, exogenous melatonin increased the expression levels of psaA and psaB genes in PSI of SD–609 and SD–902, revealing the molecular mechanism by which exogenous melatonin improves the activity of PSI under drought stress. Additionally, the cytochrome b6f complex is involved in the electron transfer between PSI and PSII. PetA and PetC are two gene transcripts related to the cytochrome b6f complex, and their expression levels in the cytochrome complex of grape leaves under drought stress decrease, indicating that electron transfer is inhibited [38]. In this study, the petA and petB genes in the cytochrome b6f complex of the two varieties were also upregulated by exogenous melatonin, suggesting that the activity of the cytochrome b6f complex and electron transfer from the PSII donor side to PQH_2_ were also improved by exogenous melatonin. The light reactions and photosynthetic electron transfer improved by melatonin provide NADPH and ATP for the dark reactions, and the improvement of photosynthetic gene expression may be an important regulatory pathway for melatonin to improve the drought stress adaptability of maize and also explain the molecular mechanism of melatonin in regulating light energy capture, energy distribution, and utilization. Moreover, compared with SD–902, treatment with exogenous melatonin had a stronger regulatory effect on the expression of important photosynthetic genes, such aspsbA, psbD, psaA, psaB, petA, andpetBin SD–609 indicating that exogenous melatonin has a more effective regulatory effect on the drought adaptability of SD–609.

## Conclusion

In conclusion, exogenous melatonin significantly enhanced the drought tolerance of maize by comprehensively improving photosynthetic performance and reinforcing the antioxidant system. It mitigates drought–induced photosynthetic inhibition by boosting both light reactions, and dark reactions. Concurrently, melatonin activates the antioxidant system, increasing enzyme activity and antioxidant content in the AsA–GSH cycle to effectively scavenge reactive oxygen species and reduce oxidative damage. The drought–tolerant genotype (SD–609) exhibited a more pronounced response to melatonin, accumulating higher levels of antioxidants and showing greater enhancement of antioxidant enzyme activity and photosynthetic gene expression. This superior regulatory capacity is the key reason for its stronger drought adaptability compared to that of the sensitive genotype (SD–902).These synergistic improvements resulted in a significant increase in dry matter production.

## Supplementary Information

Supplementary Material 1.

## Data Availability

The datasets generated and/or analyzed during the current study are available in the Shanxi Agricultural University, Jinzhong. All data generated or analyzed during this study are included in this published article files.

## Abbreviations

P_n_: Net photosynthetic rate
G_s_: Stomatal conductance
C_i_: Intercellular CO_2_ concentration
T_r_: Transpiration rate
CA: Carbonic anhydrase
PEPC: Phosphoenolpyruvate carboxylase
NADP–ME: NADP–malic enzyme
PPDK: Pyruvate phosphate dikinase
Rubisco: Ribulose–1,5–bisphosphate carboxylase/oxygenase
H_2_O_2_: Hydrogen peroxide
O_2_ .–: Superoxide anion
MDA: Malondialdehyde
AsA: Reduced ascorbate
DHA: Oxidized ascorbate
GSH: Reduced glutathione
GSSG: Oxidized glutathione
SOD: Superoxide dismutase
POD: Peroxidase
CAT: Catalase
APX: Ascorbate peroxidase
MDHAR: Monodehydroascorbate reductase
DHAR: Dehydroascorbate reductase
PSI: Photosystem I
PSII: Photosystem II
Q_A_: primary quinone acceptor of PSII
ABS/RC: Average absorbed photon flux per active PSII
DI_0_/RC: The flux of energy dissipated in processes other than trapping per active PSII
ET_0_/RC: The flux of electrons transferred from Q PQ per active PSII
ETR(I): Electron transport rate of PSI
ETR(II): Electron transport rate of PSII
F_s_: The steady–state fluorescence after light adaptation
F_m_: Maximal fluorescence of the dark–adapted state
Fm': Maximal fluorescence of the light–adapted state
V_t_: The relative variable fluorescence at time t
F_v_/F_m_: The maximal PSII photochemistry efficiency
V_k_: The relative fluorescence intensity at 300 µs
V_j_: The relative fluorescence intensity at 2 ms
V_i_: The relative fluorescence intensity at 30 ms
P_m_: The maximal P700 signal
P_m'_: The actual maximal P700 signal
P: The intermediate P700 signal
OEC: Oxygen–evolving complex
PI_ABS_: Performance index on absorption basis
PQ: Plastoquinone
TR_0_/RC: Maximum trapped exciton flux per active PSII
V_PSI_: Maximum decreasing rate of MR/MR_0_ in the range of 0.7–3 ms
V_PSII–PSI_: Maximum increasing rate of MR/MR_0_ in the range of 9–30ms
