# Supplementary material for: Exogenous melatonin on photosynthesis and physiological characteristics in maize under drought stress
Source: BMC Plant Biol. 2026 Jan 28;26:359. doi: 10.1186/s12870-026-08100-0 (PMC12924503; doi:10.1186/s12870-026-08100-0)
Supplement: Supplementary file 1 — Supplementary Material 1. [file 12870_2026_8100_MOESM1_ESM.docx]

| Table. 1S. Effects of exogenous melatonin on chlorophyll content (SPAD) and dry matter accumulation of SD–609 and SD–902 under drought stress | | | | | |
| --- | --- | --- | --- | --- | --- |
| Treatment | | SPAD Value | Dry Matter (g) | Visual Bar Chart | |
|  |  |  |  | SPAD Value | DMA Value |
| CK | SD–609 Water | 52.3 | 16.5 | SPAD: ███████████ | DMA: ███████████████ |
| CK | SD–902 Methanol | 54.1 | 17.8 | SPAD: ████████████ | DMA: █████████████████ |
| MS | SD–902 Water | 41.2 | 11.2 | SPAD: ████████ | DMA: ██████████ |
| MS | SD–609 Methanol | 43.5 | 12.5 | SPAD: █████████ | DMA: ███████████ |
| SS | SD–609 Water | 32.8 | 5.8 | SPAD: █████ | DMA: █████ |
| SS | SD–902 Methanol | 35.2 | 6.9 | SPAD: ██████ | DMA: ██████ |
| **Note**: SD–609 water: Spray distilled water on Shaandan 609; SD–609 melatonin: Spray melatonin on Shaandan 609; SD902 water: Spray distilled water on Shaandan 902; SD–902 melatonin: Spray melatonin on Shaandan 609. CK: Well-watered, soil water content at 80 % of field capacity; MS: Moderate drought stress, soil water content at 50 % of field capacity; SS: Severe drought stress, soil water content at 35 % of field capacity. Different letters indicate significant difference according to Duncan’s multiple range tests (*P* < 0.05). | | | | | |


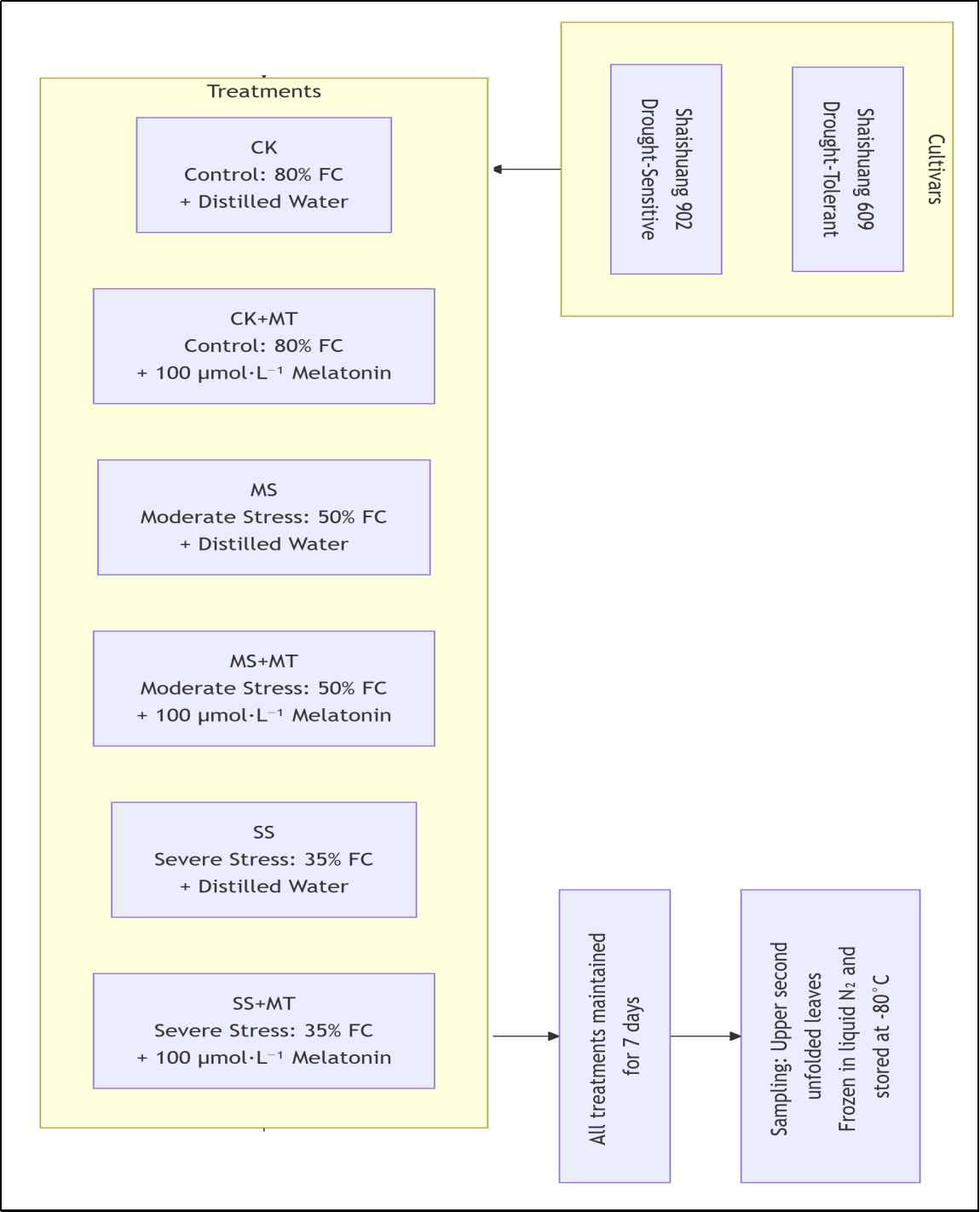


Figure.1S. Two maize cultivars (drought-tolerant Shaishuang 609 and drought-sensitive Shaishuang 902) were subjected to a factorial combination of three soil moisture levels (80%, 50%, and 35% of field capacity) and two foliar spray treatments (distilled water or 100 µmol·L⁻¹ melatonin). After maintaining the stress treatments for seven days, leaf samples were harvested for subsequent physiological and molecular analysis.


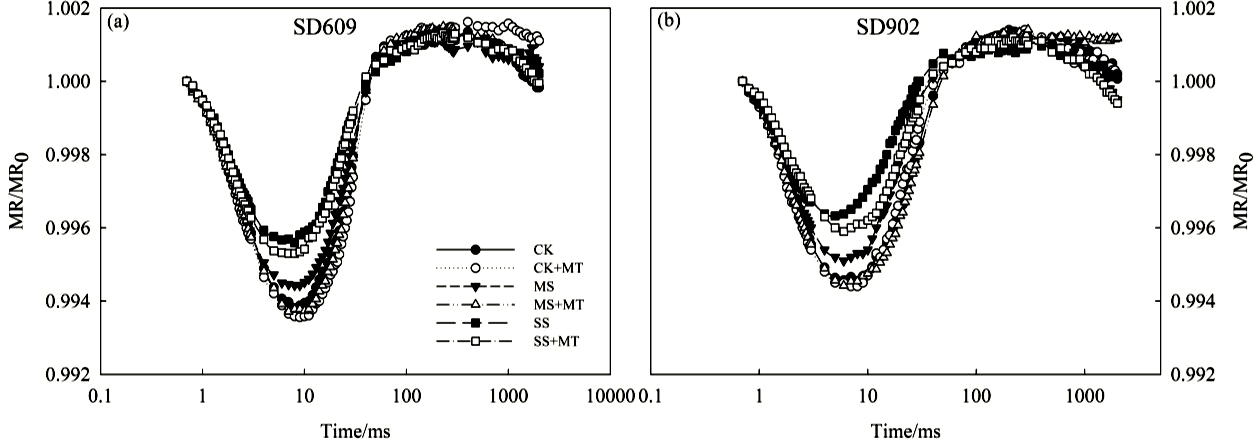


Figure.2S. Effects of exogenous melatonin on MR/MR_0_ of SD–609 and SD–902 under drought stress


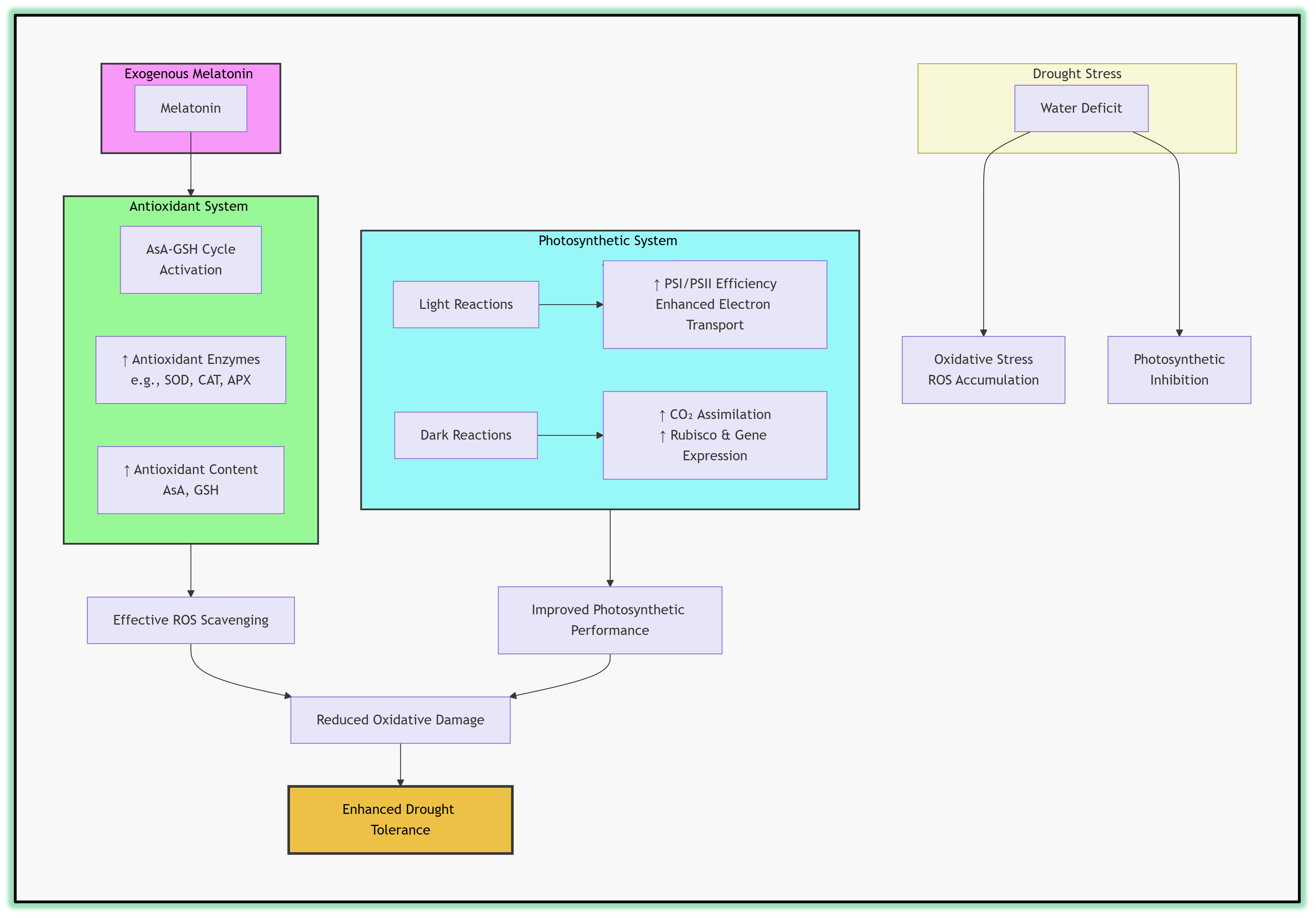


**Figure 3S. Model illustrating the role of exogenous melatonin in enhancing drought tolerance in maize and its genotypic variation.**

Under drought stress, plants experience photosynthetic inhibition and oxidative stress. Exogenous melatonin application mitigates these effects through a dual mechanism: 1) **Reinforcing the antioxidant system** by activating the AsA-GSH cycle, increasing antioxidant enzyme activity, and boosting antioxidant content, leading to effective ROS scavenging and reduced oxidative damage. 2) **Improving photosynthetic performance** by enhancing both light reactions (PSI/PSII energy conversion and electron transport) and dark reactions (CO₂ assimilation via upregulation of key enzymes and their genes).
 Comparative response of drought-tolerant (Shaandan 609) and drought-sensitive (Shaandan 902) genotypes to melatonin under drought. Data represent mean ± SE (n=5). Shaandan 609 shows a significantly greater enhancement in key physiological and biochemical parameters in response to melatonin, highlighting its superior regulatory capacity. Asterisks indicate significant differences between D and D+MT within a genotype ( **p** < 0.05, ***p** < 0.01).
